# Phytochemical Background Mediates Effects of Pyrrolizidine Alkaloids on Western Flower Thrips

**DOI:** 10.1007/s10886-018-1009-2

**Published:** 2018-09-16

**Authors:** Xiaojie Liu, Klaas Vrieling, Peter G. L. Klinkhamer

**Affiliations:** 1 Plant Ecology and Phytochemistry, Institute of Biology, Leiden University, PO Box 9505, 2300 RA Leiden, The Netherlands; 2 Modern Research Center for Traditional Chinese Medicine, Shanxi University, Taiyuan, 030006 China

**Keywords:** Liquid-liquid partition, Synergism, Antagonism, Plant defence, *Frankliniella occidentalis*, Predominant storage

## Abstract

Plants produce an extremely diverse array of metabolites that mediate many aspects of plant-environment interactions. In the context of plant-herbivore interactions, it is as yet poorly understood how natural backgrounds shape the bioactivity of individual metabolites. We tested the effects of a methanol extract of *Jacobaea* plants and five fractions derived from this extract, on survival of western flower thrips (WFT). When added to an artificial diet, the five fractions all resulted in a higher WFT survival rate than the methanol extract. In addition, their expected combined effect on survival, assuming no interaction between them, was lower than that of the methanol extract. The bioactivity was restored when the fractions were combined again in their original proportion. These results strongly suggest synergistic interactions among the fractions on WFT survival rates. We then tested the effects of two pyrrolizidine alkaloids (PAs), free base retrorsine and retrorsine *N*-oxide, alone and in combination with the five shoot fractions on WFT survival. The magnitude of the effects of the two PAs depended on the fraction to which they were added. In general, free base retrorsine was more potent than retrorsine *N*-oxide, but this was contingent on the fraction to which these compounds were added. Our results support the commonly held, though seldom tested, notion that the efficacy of plant metabolites with respect to plant defence is dependent on their phytochemical background. It also shows that the assessment of bioactivity cannot be decoupled from the natural chemical background in which these metabolites occur.

**Electronic supplementary material:**

The online version of this article (10.1007/s10886-018-1009-2) contains supplementary material, which is available to authorized users.

## Introduction

Plant metabolites play an important role in aiding the plant to cope with biotic and abiotic stresses (Fraenkel 1959; Harborne 1990*;* Hartmann 1996, 2007; Kliebenstein 2012; Wink 1988). When attempting to identify the metabolites that are responsible for a certain bioactivity in a plant, the most common approach is to isolate single metabolites and test them individually in bioassays (Hadacek 2002). This single metabolite based approach only explains a small percentage of the activity in an extract. This is because other metabolites in the mixture also play a role, either directly or through synergistic or antagonistic effects. These interactive effects have not received much attention in the literature. This is surprising given that plant metabolites always occur in diverse mixtures with thousands of other metabolites they can interact with (Williamson 2001; Wink 2003). Because of these potential interactions, the bioactivity of metabolite mixtures is often different from the sum of the effects of the individual metabolites. Synergy between metabolites can provide plants with a more efficient defence against herbivores if the combined activity is superior to the sum of activities of the individual metabolites (Berenbaum and Zangerl 1993; Nelson and Kursar 1999). The contribution of single metabolites to plant fitness will be underestimated if their effect is determined separately from the natural chemical background in which they occur. Interactions between plant metabolites present a great challenge to researchers in terms of the number of metabolites and even more so in terms of the number of potential combinations that need to be tested. The latter may be the main reason why interactions between metabolites have not been widely studied.

To explain the chemical diversity within a certain class of metabolites, previous studies mostly focus on combinations of two or more metabolites of that chemical class (Diawara et al. 1993; Dyer et al. 2003; Liu et al. 2017a; Richards et al. 2010, 2012; Smith et al. 2001; Whitehead and Bowers 2014). However, plants contain metabolites of different classes (Berenbaum and Neal 1985; Guillet et al. 1998; Liu et al. 2017b; Neal 1989; Nelson and Kursar 1999; Nuringtyas 2014). Given the enormous number of metabolites in a single plant, it is impossible to evaluate all possible combinations. In addition, interactions may occur among unidentified or even unknown metabolites, which represent a great part of the total amount of plant metabolites (Trethewey 2004). In view of these facts, the question is how to measure the interactions between plant metabolites without prior knowledge about which specific metabolites are involved and what their potential mode of action is. A top-down approach using fractions, rather than combinations of individual metabolites provides an alternative starting point.

In this study, we used two different approaches to investigate the interactions between plant metabolites on insect herbivores. First, we tested combinations of polar fractions of plant extracts. If interactions between metabolites determine the bioactivity, separating the interacting metabolites into different fractions may result in the loss of the activity. This should be restored again if the fractions are recombined in their original proportions. Second, we tested the effect of plant metabolites in isolation and in the background of plant extracts or fractions. Plant extracts and fractions can be regarded as the natural phytochemical background in which plant metabolites occur. An increase in the bioactivity of a compound when added to extracts or fractions would indicate the presence of synergistic interactions among plant compounds. A decrease would indicate antagonistic interactions.

We used *Jacobaea vulgaris* and its pyrrolizidine alkaloids (PAs) as a study system. *Jacobaea* species are characterized by their PAs, which play a pivotal role in their defence. Negative effects of PAs on mammalian herbivores (e.g. Fu et al. 2001; EFSA report 2011; Trigo 2011), generalist insect herbivores (e.g. de Boer 1999; Dreyer et al. 1985; Domínguez et al. 2008; Kostenko et al. 2013; Liu et al. 2017a; Macel 2011; Reina et al. 2001; Siciliano et al. 2005; Wei et al. 2015) and pathogens (e.g. Joosten and van Veen 2011; Rubiolo et al. 1992) have been reported. In plants, PAs occur mostly as PA *N*-oxides (Hartmann et al. 1989) with some jacobine-like PAs occurring up to 50% as free base in *J. vulgaris* (Joosten et al. 2011). Generally the PA *N*-oxides are less active against insect herbivores than the corresponding free bases (Dreyer et al. 1985; Liu et al. 2017a; Macel et al. 2005; Nuringtyas et al. 2014; van Dam et al. 1995). However the bioactivity was reversed when *N*-oxides and free base PAs were combined with chlorogenic acid (CGA). CGA showed synergistic interactions with PA *N*-oxides and antagonistic interactons with free base PAs (Liu et al. 2017b). These results clearly showed that bioactivity of individual metabolites may differ from that their bioactivity in concert with other metabolites. It emphasises the importance of taking into account the interactions between plant metabolites when investigating their ecological functions.

Retrorsine and retrorsine *N*-oxide were tested in the current study because we already found retrorsine to be the most active against thrips larvae. Retrorsine *N*-oxide had no effect on thrips survival and it showed a strong interaction with chlorogenic acid (Liu et al. 2017a, b). Furthermore, retrorsine and retrorsine *N*-oxide occur in low quantities in *Jacobaea* plants so that interference with natural retrorsine and retrorsine *N*-oxide present in the plant material is minimal.

The purpose of this study is twofold. First, we asked the more general question if interactions between metabolites are likely to play a role in the defence of *J. vulgaris* against western flower thrips (WFT) (*Frankliniella occidentalis*). We tested the effects of a methanol extract of *J. vulgaris* shoots, the five solvent-solvent fractions of the methanol extract and the re-combined fractions on WFT performance. Secondly, we asked if PAs interact with other plant metabolites. We tested free base retrorsine (further referred to as retorsine) and retrorsine *N*-oxide alone and in combination with plant fractions. We addressed the following questions: Does the methanol extract of *Jacobaea* reduce WFT survival? Is the acitivity against WFT maintained after fractionation of the methanol extract? Is the effect on WFT survival restored if fractions are recombined again? How do plant fractions interact with retrorsine and retrorsine *N*-oxide on WFT survival? Are the retrorsine and retrorsine *N*-oxide equally effective in the background of the fractions?

## Materials and Methods

### Chemicals and Plant Materials

**Fig. 1:**
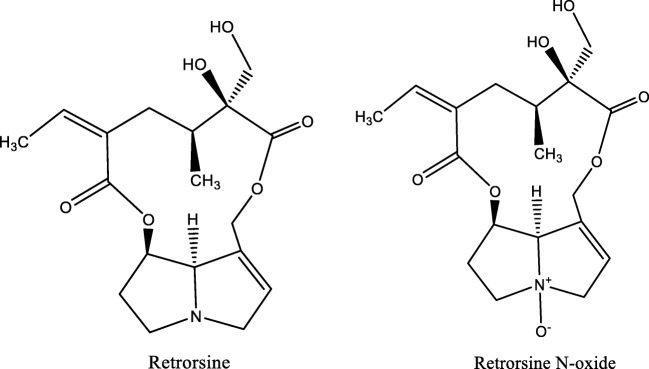
Chemical structures of retrorsine and retrorsine *N*-oxide

Retrorsine and retrorsine *N*-oxide were purchased from Sigma (St. Louis, MO, USA). The chemical structures are provided in Fig. 1.

One hundred F2 genotypes of a cross between *Jacobaea vulgaris* and *Jacobaea aquatica* were planted in a garden in Lisse (52° 25′ 12” N, 4° 54′10″ E, The Netherlands) and grown for 17 months and harvested in March 2013. All plants were separated into shoots and roots, and were dried in an oven at 50 °C, milled to a fine powder, then stored in an air-tight container and kept at room temperature until further use. The shoots of all plants were pooled.

### Extraction of Jacobaea Shoots

Fifty grams of powdered *Jacobaea* shoot material was 3 times extracted for one hour with 80% methanol containing 0.1% formic acid (3 × 3 l) using a speed extractor (Büchi E-916, Büchi Labortechnik, Flawil, Switzerland) at 30 °C and 50 bar. The three crude methanol extracts were combined, evaporated under reduced pressure and re-dissolved in 100 ml of water. The aqueous extract was successively extracted with *n*-hexane (3 × 100 ml), chloroform (CHCl_3_) (3 × 100 ml), ethyl acetate (EtOAc) (3 × 100 ml) and *n-*butanol (*n*-BuOH) (3 × 100 ml). Removal of the solvents under reduced pressure yielded the *n*-hexane, CHCl_3_, EtOAc, *n*-BuOH fractions and the residual H_2_O fraction.

### WFT Bioassay

Second instar larvae of the western flower thrips (WFT), *Frankliniella occidentalis* Pergande (Thripidae), were obtained from a lab culture reared on chrysanthemum flowers in a growth chamber at 23 °C, 12 h L/D photoperiod, 60% RH. WFT bioassays were conducted in adapted 96-well plates filled with 55 μl test solution covered with Parafilm™ (Liu et al. 2017a). Single second instar larvae of WFT were placed into the lids of an 8 cap flat- strip. Each lid was sealed with Parafilm™ and then placed on top of the 96 well plates. The 96 wells plates were placed upside down so that WFT larvae were able to feed from the offered test solutions by piercing through the Parafilm™ (Fig. S1). The plates were randomly placed into a growth chamber with standard WFT rearing conditions (L:D, 12:12, 23 °C).

The test solution consisted of 10% fructose and a phosphate buffer (NaH_2_PO_4_ and Na_2_HPO_4_, 40 mM, pH 7). The total methanol extract, five fractions and the combinations of fractions with PAs were dissolved in methanol and added to this solvent so that the final concentration of methanol was always 3%. The negative control was the test solution with 3% methanol. There were two positive controls: empty wells (without solution) to verify that WFT larvae could not survive without feeding and a solution with the insecticide abamectine (50 μg/ml).

All the wells in a column in a 96 wells plate received the same treatment. One treatment group therefore consisted of 8 replicates (wells). Each treatment was added to columns on 4 different 96 well-plates, thus yielding 32 wells for each treatments (Fig. S2). After 5 days, the numbers of surviving larvae were counted with a stereo microscope (100X). Mortality rate was calculated as the number of dead larvae in a treatment in one replicate divided by the total number of larvae in that treatment. WFT survival rates were calculated as 1 minus WFT mortality rate and then corrected for WFT survival in the controls as indicated by formula 1 (see below).

### *WFT survival on the Jacobaea* Methanol Shoot *extract and Its five fractions*

The concentrations of the methanol extract and the five fractions were expressed as the equivalent amount of dried plant material from which they were derived: 0.02, 0.04, 0.06 and 0.08 g plant mass equivalents/ml. One gram of plant mass yielded 194.0, 3.0, 5.7, 7.4, 22.8 and 128.9 mg of the methanol extract, *n*-hexane, the CHCl_3_, the EtOAc, the *n*-BuOH and the H_2_O fractions, respectively.

The bioassays with the methanol extract of *Jacobaea* shoots and the five fractions thereof were carried out once consisting of 32 thrips larvae per treatment across four 96-well plates. Of the 12 columns of the 96-well plate, seven columns were filled with seven different treatments and five columns were left empty (Fig. S2). The seven treatments included the negative control (test solution), four concentrations of the methanol extract and two positive controls (empty wells and abamectin) as shown in Fig. S2. Each treatment consisted therefore of 4 × 8 = 32 wells. In the same way the five fractions were tested.

### WFT Survival on the Recombined Fractions

The five fractions (*n*-hexane, CHCl_3_, EtOAc, *n*-BuOH and H_2_O) were recombined in the original proportion to reconstitute the original methanol extracts. The combined fractions were tested at the same concentrations as the methanol extracts. The concentrations of the fractions were exactly five times more concentrated than the original concentration. Thus, after combining the five solutions in equal amounts the final concentration was equal to the undiluted original methanol extract. In practice, 400 μl solution from each fraction was added to obtain the recombined extract at the highest concentration, i.e. 0.08 g/ml plant mass equivalents. Afterwards, a series of concentrations was obtained by dilution. The recombined fractions were tested at 0.02, 0.04, 0.06 and 0.08 g/ml plant mass equivalents. The bioassay of the re-combined five fractions was carried out as indicated above.

### The PA Content in Plant Fractions Measured by LC-MS/MS

Liquid chromatography-tandem mass spectrometry (LC-MS/MS) analyses of PAs of the plant fractions were conducted based on a protocol described by Cheng et al. (2011). Prior to analysis, 10 μl of each fraction or sub-fraction in DMSO was diluted with 1 ml of water and transferred to an HPLC vial. Analysis was conducted on an Acquity UPLC system coupled to a Quattro Premier XE tandem mass spectrometer (Waters, Milford, MA, USA) operated in positive electrospray mode. Separation of the PAs was accomplished on a BEH C18 150 × 2.1 mm, 1.7 μm, UPLC column (Waters) by using an acetonitrile/water/6.5 mM ammonia gradient, from 0 to 50% acetonitrile in 12 min. Column temperature was set at 50 °C and the flow was at 400 μl/min. The PAs were quantified using external standard calibration prepared from a blank extract spiked with PA standards (range: 0–500 ng/ml). The limit of quantification for individual PAs in the fractions was approx. 0.5 μg/g dry plant material.

The five fractions were all found to contain PAs. The majority of the PAs were detected in the CHCl_3_ fraction (67%, which is equal to 0.24 mM PAs) (Fig. S3). The *n*-BuOH fraction contained 24%, the H_2_O fraction 5% and the EtOAc fraction 3% and the *n*-hexane fraction 0.2% of the total PA content (Fig. S3). The WFT survival rates on the fractions were not correlated with their PA content (*r* = 0.03, *n* = 5, NS).

### WFT Survival on the Jacobaea Methanol Shoot Extract and Five Fractions Spiked with Retrorsine and Retrorsine N-oxide

Retrorsine and retrorsine *N*-oxide were combined with the methanol extract and either one of the five fractions yielding 12 combinations including the bioassays with single metabolites. The final concentrations of the extracts were 0, 0.01, 0.05, and 0.09 g/ml plant mass equivalents and retrorsine and retrorsine *N*-oxide with concentrations of 0, 1.4 and 7.0 mM. The doses of retrorsine and retrorsine *N*-oxide represent 1.0 and 5.0 times the total PA concentration of fresh *J. vulgaris* plants.

For the combination tests, experiments were conducted twice on different days, giving two independent estimates of WFT survival for the analysis of variance. Each bioassay was performed four times on two 96-well plates with 14 columns filled with 14 different treatments, and 10 columns left empty. The 14 treatments included: the negative control, 12 treatment groups (three concentrations of the methanol extract/fraction, two concentrations of a PA and six combinations of PAs and methanol extract/fraction) and two positive controls (empty wells and abamectin). Each treatment therefore consisted of 32 wells. The complete bioassay was repeated on a different day so that we obtained two independent estimates of 32 wells. Experiments were conducted twice to have two independent estimates of WFT survival rates (*n* = 2) for analysis of variance.

### Statistical Analysis

WFT survival rates were log-transformed to obtain a linear relationship with concentration. We calculated the slopes of the regression lines and their 95% confidence intervals (CIs) to compare the effects of the extract and different fractions. If two slopes had non-overlapping 95% confidence intervals they were assumed to be significantly different at *P* < 0.05.

### Correcting for Survival in the Negative Control

In order to examine the interaction effect of combinations of metabolites and extracts/fractions, we constructed a null model, in which the expected WFT survival of a combination of metabolites (or/extracts or fractions) is the product of the survival of the single metabolites assuming there are no interaction effects (Liu et al. 2017b).

In a similar manner, we corrected for survival in the negative control. The survival (S_X + NC_) after the application of metabolite X in a test solution can be calculated as the combined effect on survival of the test solution (S_NC_) and that of metabolite X (S_X_).

1 $$ {\displaystyle \begin{array}{l}{\mathrm{S}}_{\mathrm{X}+\mathrm{NC}}={{\mathrm{S}}_{\mathrm{NC}}}^{\ast }\ {\mathrm{S}}_{\mathrm{X}\kern1em }\mathrm{or}:\\ {}{\mathrm{S}}_{\mathrm{X}}={\mathrm{S}}_{\mathrm{X}+\mathrm{NC}}/{\mathrm{S}}_{\mathrm{NC}}\end{array}} $$

### Estimating Antagonistic and Synergistic Effects on WFT Survival of Recombining the Five Fractions

Analogous to the above we calculated the expected WFT survival of the recombined fractions based on the survival of the five fractions under the assumption that no interaction occurs (S_recF, exp_) by:

2 $$ {\mathrm{S}}_{\mathrm{recF},\exp }={{\mathrm{S}}_{n\hbox{-} \mathrm{hexane}}}^{\ast }{{\mathrm{S}}_{\mathrm{chloroform}}}^{\ast }{{\mathrm{S}}_{\mathrm{EtOAc}}}^{\ast }{{\mathrm{S}}_{n\hbox{-} \mathrm{BuOH}}}^{\ast }{\mathrm{S}}_{\mathrm{aqueous},} $$

Where S_*n*-hexane_, S_chloroform_, S_EtOAc_, S_*n-*BuOH_, S_aqueous_ are the observed WFT survival rates of the different fractions. The interaction effect on survival when combining the fractions (S_F*F_) can be calculated as the observed survival after recombing all fractions (S_recF, obs_) divided by the expected survival if no interaction occurs (S_recF, exp_)

3 $$ {\mathrm{S}}_{\mathrm{F}\ast \mathrm{F}}={\mathrm{S}}_{\mathrm{recF},\mathrm{obs}}/{\mathrm{S}}_{\mathrm{recF},\exp } $$

Note that also the survival rates are corrected for the negative control according to formula 1. S_F*F_ indicates the interaction between fractions.

### Estimating Antagonistic and Synergistic Effects on WFT Survival

The survival of WFT for the combination of a PA and an extract/fraction (S_PA + F_) results from the survival after application of the PA (S_PA_), the survival after application of the extract/fraction (S_F_), and their interaction (S_PA*F_). The effect of the interaction on survival can be calculated by:

4 $$ {\displaystyle \begin{array}{l}{\mathrm{S}}_{\mathrm{PA}+\mathrm{F}=}{\mathrm{S}}_{\mathrm{PA}\ast }{\mathrm{S}}_{\mathrm{F}\ast }{\mathrm{S}}_{\mathrm{PA}\ast \mathrm{F}}\kern1em \mathrm{or}\\ {}{\mathrm{S}}_{\mathrm{PA}\ast \mathrm{F}}={\mathrm{S}}_{\mathrm{PA}+\mathrm{F}}/\left({{\mathrm{S}}_{\mathrm{PA}}}^{\ast }\ {\mathrm{S}}_{\mathrm{F}}\right)\end{array}} $$

In which S_PA + F_ is the observed survival in experiments with the combination of PA and an extract/fraction while S_PA_ and S_F_ are the observed WFT survival in experiments with the PA and fraction only. S_PA*F_ denotes the interaction effect of PA concentration and fractions. When S_PA*F_ equals one the effects of S_PA_ and S_F_ are multiplicative and there is no interaction between them. If the interaction effect S_PA*F_ < 1 there is a synergistic interaction and if S_PA*F_ > 1 there is an antagonistic interaction.

As each experiment was repeated twice, two independent estimates of the interaction effect S_PA*F_ were obtained. The interaction effect S_PA*F_ was calculated for all combinations and was expressed as mean value ± standard error of the mean (SE). To estimate if an interaction effect S_PA*F_ deviates significantly from one, a two-way analysis of variance (ANOVAs) was used with the concentration of the PAs and fractions as factors. As dependent variables the two estimates of S_PA*F_ -1 = [S_PA + F_/ (S_PA_ * S_F_)] -1 were used for all combinations of traits. Therefore, if the intercept of the two-way ANOVA is significantly deviating from zero it indicates that S_PA*F_ -1 is deviating from zero and hence that S_PA*F_ is significantly deviating from one. If the interaction effect S_PA*F_ is <1 it indicates a synergistic interaction and if it is >1 it indicates an antagonistic interaction.

A four-way analysis of variance (ANOVA) was performed with two PAs (retrorsine and retrorsine *N*-oxide), PA concentration, five fractions and the concentration of the fraction as factors with the interaction effect S_PA*F_ as a dependent variable.

### Comparison of the Interaction Effects of Retrorsine and Retrorsine N-oxide when Combined with Plant Fractions

To compare the interaction effects of retrorsine and retrorsine *N*-oxide on WFT with that of plant fractions, we plotted the value of ∆ WFT mortality against the concentrations of the PAs and fractions. ∆ WFT mortality is the average mortality of retrorsine mixed with a plant fraction minus the average mortality of retrorsine *N*-oxide mixed with the same plant extract/fraction. When ∆ WFT mortality >0 retrorsine is more potent when ∆ WFT mortality <0 retrorsine *N*-oxide is more potent.

The standard deviation (σ) of the ∆ WFT mortality was calculated by Eq. (5), where σ (retrorsine *N*-oxide) and σ (retrorsine) are the standard deviation of the WFT mortality of retrorsine *N*-oxide mixed with a fraction and the standard deviation of the WFT mortality of retrorsine mixed with a plant fraction.

5 $$ \boldsymbol{\upsigma} \left(\Delta\;\mathbf{WFT}\;\mathbf{mortality}\right)=\sqrt{\boldsymbol{\upsigma} {\left(\mathbf{retrorsine}\;\boldsymbol{N}-\mathbf{oxide}\right)}^{\mathbf{2}}+\boldsymbol{\upsigma} {\left(\mathbf{retrorsine}\right)}^{\mathbf{2}}} $$

The 95% confidence intervals (CIs) of ∆ WFT mortality were then estimated by Eq. (6).

6 $$ \mathbf{95}\%\mathbf{CIs}={\frac{\boldsymbol{\sigma} \left(\Delta\;\mathbf{WFT}\;\mathbf{mortality}\right)}{\sqrt{\mathbf{n}}}}^{\ast}\mathbf{1.96} $$

With σ (∆ WFT mortality) being the standard deviation from Eq. (5) and n being number of replicates for each combination (*n* = 2). 95% CIs are used to determine if ∆ WFT mortality is significantly deviating from zero.

All statistical analysis were performed using SPSS software for Windows (version 21.0; SPSS Inc., Chicago, IL).

## Results

### WFT Survival on the Jacobaea Methanol Shoot Extract and Five Fractions

WFT survival rates in the negative control was on average 0.81 ± 0.02 while the positive control with the insecticide abamectine (50 μg/ml) solution showed an average WFT survival rate of 0.10 ± 0.02. The control with empty wells, to verify that WFT cannot survive without feeding, had an average survival rate of 0.04 ± 0.01.

**Fig. 2:**
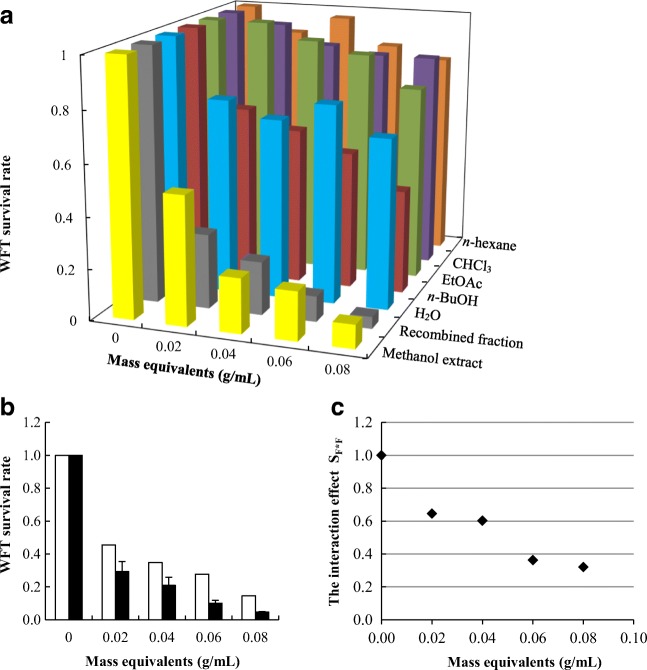
Western flower thrips (WFT) survival rates of the five fractions (S_F_) and the recombined fractions (**a**), the expected survival assuming no interaction (white bars), the observed survival of the re-combined fractions (black bars) (**b**), and the interaction effect among fractions (S_F*F_) (**c**) at the five tested concentrations of *Jacobaea* mass equivalents

With increasing concentrations of the methanol extract, WFT survival rates decreased (Fig. S4). After fractionation, the effect of either individual fraction was significantly lower than that of the methanol extract (Fig. 2a). The methanol extract resulted in the steepest negative slope (Table 1) between log-transformed WFT survival rate and concentration (Fig. S4).

**Table 1:** The slopes of the regression of log-transformed survival ± their 95% confidence intervals (CIs) of log survival of 2^nd^ instar Western flower thrips (*Frankliniella occidentalis*) on the concentrations of the methanol extract of *Jacobaea* or fractions derived from the methanol extract and the estimated LD_50_

Plant extract/fractions	R^2^	F_1,4_	*P* value	Slope ± 95% CIs	Estimated LD_50_ (g/mL)
Methanol extract	0.98	168.0	*P* < 0.001	−13 ± 3.2^a^	0.02
*n*-hexane fraction	0.72	2.1	NS	−0.9 ± 2.6^bc^	0.34
CHCl_3_ fraction	0.90	27.3	P < 0.05	−0.9 ± 0.6^b^	0.33
EtOAc fraction	0.86	18.6	*P* < 0.05	−1.4 ± 1.0^b^	0.22
*n*-BuOH fraction	0.95	60.4	*P* < 0.01	−4.4 ± 1.8^c^	0.07
H_2_O fraction	0.49	5.5	NS	−1.7 ± 2.3^bc^	0.17

R^2^ is explained variance, *F* indicates the *F* value of the corresponding analysis of variance. Letters behind the slopes indicate significant differences between slopes

NS = Not significant

All fractions affected WFT survival rates but to different extents. The rank of the slopes (± 95% CIs) was *n*-BuOH fraction, H_2_O fraction, EtOAc fraction, CHCl_3_ fraction and *n*-hexane fraction (Table 1). Based on the 95% CIs of the slopes, log-transformed WFT survival rates significantly differed between the *n*-hexane/H_2_O fractions, the EtOAc/CHCl_3_ fractions and the *n*-BuOH fraction (Fig. S4, Fig. S5; Table 1). Note that only for the *n*-BuOH, the EtOAc and the CHCl_3_ fractions we found a significant concentration-dependent decrease of log-transformed WFT survival rates.

The recombined methanol extract of the five fractions in their original mass ratios showed a similar WFT survival rate as the original methanol extract (Fig. 2a). WFT survival rate of the recombined extract was lower than the expected survival rate based on any of the individual fractions indicating that a synergistic interaction between the fractions on survival was present (Fig. 2b, c).

### The Interaction between Retrorsine, Retrorsine N-oxide and Jacobaea Shoot Fractions on WFT Survival

**Fig. 3:**
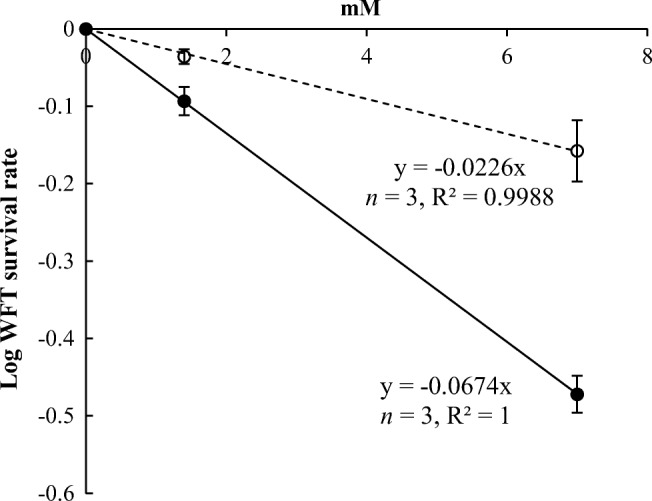
Log-transformed survival rates of 2^nd^ instar Western flower thrips (WFT) (*Frankliniella occidentalis*) against the concentration of retrorsine (solid dots) and retrorsine *N*-oxide (open dots)

Retrorsine and retrorsine *N*-oxide showed a significant dose-dependent effect on WFT survival rates (Fig. 3).

**Fig. 4:**
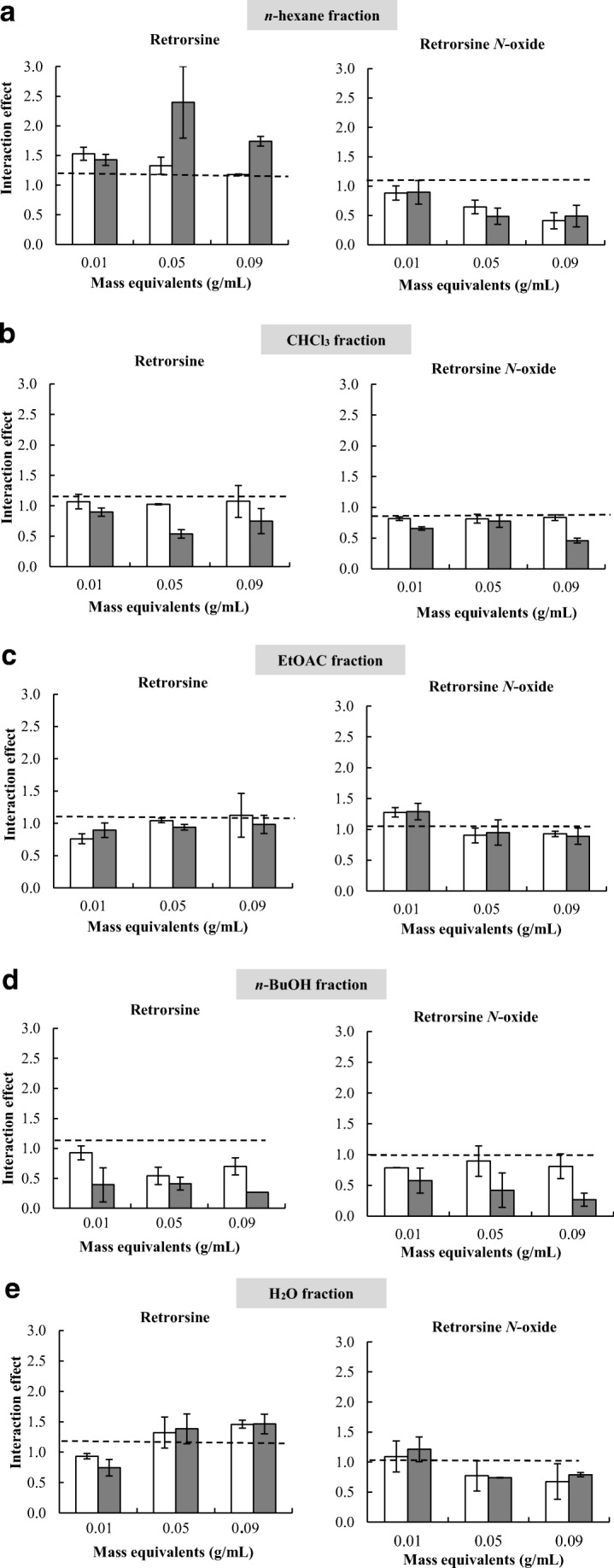
Magnitude of the interaction effect (mean ± SE) against concentration of shoot fractions expressed as *Jacobaea* mass equivalents (g/mL) for PA at 1.4 mM (white bars) and at 7.0 mM (grey bars). The combination of *n*-hexane fraction (**a**), CHCl_3_ fraction (**b**), EtOAc fraction (**c**), *n*-BuOH fraction (**d**) and H_2_O fraction (**e**) with retrorsine (**on the left**) and with retrorsine *N*-oxide (**on the right**). Deviation of the interaction effect S_PA*F_ from the dashed line indicates a synergistic (<1) or an antagonistic effect (>1). Two-way ANOVAs were used to analyze whether the overall interaction effect of PA concentration and fractions (S_PA*F_) deviated from one (Table S1). * The survival rate of WFT in one replicate of the combination of *n-*BuOH and retrorsine at 7.0 mM was zero, therefore it was considered a missing value

Retrorsine showed a significant interaction effect of PA concentration and fraction with *n*-hexane, *n*-BuOH and H_2_O fraction on WFT survival rates (left graphs in Fig. 4a, d, and e, Table S1) The *n*-BuOH fraction combined with retrorsine decreased WFT survival rates synergistically (Fig. S6D). In contrast, the combinations of the *n*-hexane and the H_2_O fractions with retrorsine increased WFT survival rate antagonistically (left graphs in Fig. 4a, e, Fig. S6, Table S1). No significant interaction effect of PA concentration and fraction was observed for the combination of the CHCl_3_ fraction and the EtOAc fraction with retrorsine (left graphs in Fig. 4b, c, Fig. S6, Table S1). For the H_2_O fraction, the ANOVA showed that its concentration affected the strength of the interaction (Table S1). For the *n*-BuOH fraction, the effect of concentration was marginally significant. In the case of the *n*-hexane fraction, the strength of the antagonistic effect was independent from the concentrations of the two components. Although for the combination of the CHCl_3_ fraction and retrorsine the intercept was not significant, the retrorsine concentration had a significant effect. At 7 mM a synergistic interaction effect between retrorsine and the CHCl_3_ fraction was present (left graphs in Fig. 4a).

For retrorsine *N*-oxide, the interaction effects of PA concentration and fraction were always lower than one, except for the EtOAc, and thus indicative of a synergistic interaction effect of PA concentration and fraction (right graphs in Fig. 4, Table S2). The synergistic interaction effects of retrorsine *N*-oxide with the *n*-hexane fraction, the CHCl_3_ fraction and *n*-BuOH fraction were statistically significant (Table S2). The ranking of the strength of the interaction effects of PA concentration and fractionwas: retrorsine *N*-oxide + *n*-BuOH (S_PA*F_ = 0.63 ± 0.09), retrorsine *N*-oxide + *n*-hexane (S_PA*F_ = 0.64 ± 0.07), retrorsine *N*-oxide + CHCl_3_ (S_PA*F_ = 0.72 ± 0.04), retrorsine *N*-oxide + H_2_O (S_PA*F_ = 0.88 ± 0.09), and retrorsine *N*-oxide + EtOAc (S_PA*F_ = 1.04 ± 0.07) (right graphs in Fig. 4).

The interaction effect of PA concentration and fraction was lower than 1 in 26 out of 30 combinations (right graphs in Fig. 4). The strongest effect was found for the interaction between retrorsine *N*-oxide and the *n*-BuOH fraction at 0.09 g/ml which lead to S_PA*F_ = 0.27 (right graphs in Fig. 4d, Fig. S7). The latter means that the combination leads to an additional mortality of 73% (1–0.27) on top of the effect of the compounds alone.

For both retrorsine and retrorsine *N*-oxide, the interaction effect between the PA concentration and the fraction were fraction-specific and PA-specific (Table 2). There was no overall effect of PA concentration or fraction. Instead, the effects of PA concentration and fraction mutually depended on each other. This is reflected by the significant interaction terms of the ANOVA (Table 2).

**Table 2:** Four-way analysis of variance (ANOVA) with PAs, fractions, PA concentration and fraction concentration as factors with the interaction effect S_PA*F_ minus one as a dependent variable

Factors	*df*	F	P
Intercept	1, 118	14.3	< 0.001
PAs	1, 118	30.7	< 0.001
Fractions	4, 118	16.5	< 0.001
PA concentration	1, 118	3.0	NS
Fraction concentration	2, 118	1.2	NS
PAs * Fractions	4, 118	17.0	< 0.001
PAs * PA concentration	1, 118	0.4	NS
PAs * Fraction concentration	2, 118	7.4	< 0.01
Fractions * PA concentration	4, 118	5.5	< 0.01
Fractions * Fraction concentration	8, 118	0.7	NS
PA concentration * Fraction concentration	2, 118	0.4	NS
PAs * Fractions * PA concentration	4, 118	1.8	NS
PAs * Fractions * Fraction concentration	8, 118	2.6	< 0.05
PAs * PA concentration * Fraction concentration	2, 118	1.1	NS
Fractions * PA concentration * Fraction concentration	8, 118	0.6	NS
PAs * Fractions * PA concentration * Fraction concentration	8, 118	1.1	NS

PAs tested are retrorsine and retrorsine *N*-oxide. The fractions are *n*-hexane, CHCl_3_, EtOAc, *n*-BuOH and H_2_O fraction of a methanol extract from *Jacobaea*. Each combination was tested in two independent bioassays. A significant intercept indicates a synergistic or antagonistic interaction

NS = Not significant

### Comparison of the Interaction Effects between Retrorsine and Retrorsine N-oxide and Jacobaea Shoot Fractions

**Fig. 5:**
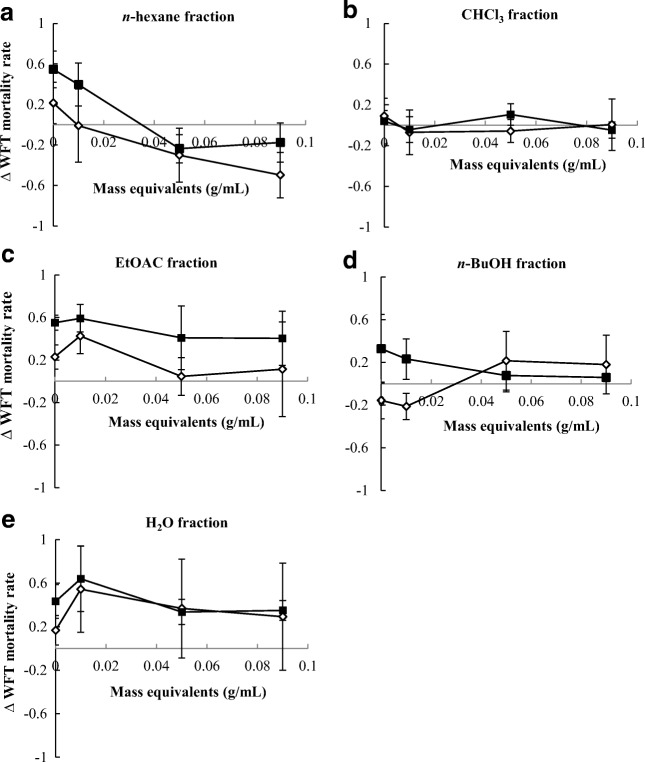
∆ mortality rate of Western flower thrips (WFT) against *Jacobaea* mass equivalents (g/mL) (mean ± 95% confidence interval) of different fractions. ∆WFT mortality rate is the difference between WFT mortality rate on a diet with retrorsine and a particular fraction and WFT mortality rate on a diet with retrorsine *N*-oxide and the same fraction (**a**) *n*-hexane fraction, (**b**) CHCl_3_ fraction, (**c**) EtOAc fraction, (**d)***n*-BuOH fraction, (**e**) H_2_O fraction. Open diamonds: PA concentration of 1.4 mM; Solid squares: PA concentration of 7 mM

We compared WFT mortality for retrorsine and retrorsine *N*-oxide by calculating the difference in WFT mortality between the two experiments, (∆WFT mortality). Firstly, retrorsine and retrorsine *N*-oxide alone resulted in a positive ∆WFT mortality indicating a lower WFT survival on retrorsine compared to retrorsine *N*-oxide (Fig. 5). In general, ∆WFT mortality was higher at a PA concentration of 7.0 mM. For the EtOAc fraction, *n*-BuOH fraction and H_2_O fraction we found that retrorsine in combination with these fractions was more detrimental to WFT than the retrorsine *N*-oxide. For the combination with the CHCl_3_ fraction no pronounced differences were observed between the combination with retrorsine and retrorsine *N*-oxide (Fig. 5b). In the case of the *n*-hexane fraction at plant concentration zero and 0.01 plant mass equivalents per ml extract, retrorsine was more toxic. At higher *n-*hexane extract concentrations retrorsine *N*-oxide was more toxic (Fig. 5a).

## Discussion

We found that WFT survival rate was affected by antagonistic and synergistic interactions between plant metabolites. Firstly, we showed that fractionation led to reduced WFT mortality on fractions compared to the original methanol extract. Re-combining the fractions in their original proportions restored the effect on WFT survival which demonstrated significant synergistic interactions between fractions on WFT survival. We then showed that adding retrorsine and retrorsine *N*-oxide to the five fractions resulted in synergistic and antagonistic interactions on WFT mortality. The current findings also suggested that retrorisne is overall more potent than its *N*-oxide, but the effect is contingent on the background chemistry of the fraction the individual PAs are tested in.

### Fractionation Weakened the Activitity of the Methanol Extract of Jacobaea Significantly with Respect to WFT Survival while Re-combination Restored the Orginial Activity

The methanol extract of the *Jacobaea* leaves significantly decreased WFT survival rates. This was even true at concentrations that were much lower than the plant concentration. Upon further fractionation of the methanol extract, most of the activity was lost. The effects of individual fractions on WFT survival rates were significantly lower than that of the methanol extract. Such a loss of activity may be due to the loss of active constituents, e.g. chemical conversion, during the fractionation. However, after recombining the fractions, the WFT survival rate was even somewhat lower than the original methanol extract. Overall we can conclude that the loss of activity upon fractionation was not due to the loss of active compounds, but resulted from the loss of synergistic interactions.

The five fractions of the methanol extract differed in their effects on WFT survival. By increasing solvent polarity each fraction contained different metabolites (Sasidharan et al. 2011). Metabolites with low polarity (e.g. essential oils) are extracted by the non-polar solvent *n*-hexane (polarity index of 0.1), while moderately polar solvents such as CHCl_3_ or EtOAc (polarity index of 4.1) mainly extract steroids, alkaloids, etc. Water, the most polar solvent (polarity index of 10.2) is effective in extracting the metabolites with higher molecular weights such as proteins, glycans, etc. (Cos et al. 2006; Anupam et al. 2012).

### Plant Fractions Significantly Interacted with Retrorsine and Retrorsine N-oxide both Antagonistically and Synergistically

In this study, we found PAs in all fractions. However, the order of the PA content of the fractions was not consistent with WFT survival (Figs. S4 and S5), suggesting that besides PAs, other metabolites also contributed directly or indirectly, to WFT mortality. The synergistic interaction between PAs and fractions on WFT survival rates are both PA and fraction specific. For instance, the strength of the interaction effect of the *n*-BuOH fraction with PAs was stronger than that of the CHCl_3_ fraction. This is despite the fact that the natural PA amount was about three times higher in the CHCl_3_ fraction than in the *n*-BuOH fraction. Again, these results suggest that interactions between PAs and other metabolites may dominate the overall synergistic effects between fractions and PAs on WFT survival. The results suggest that using only single metabolites to study bioactivity underestimates the bioactivity of these metabolites in their natural phytochemical background as is often reported in phytochemical studies (Herrera and Amor 2011; Inui et al. 2012; Labuschagne et al. 2012; Williamson 2001).

### The Free Base and the N-oxide Are Not Equally Effective in the Background of the Fractions

We previously studied the interaction between PAs and CGA on WFT survival. We found antagonistic effects between retrorsine and CGA on WFT survival while we found synergistic effects between retrorsine *N*-oxide and CGA (Liu et al. 2017b). Here, we found synergistic interactions between both retrorsine *N*-oxide and retrorsine and the *n*-BuOH fraction, even though this fraction contained a high concentration of CGA (Fig. S8). Apparently other metabolites masked or even overrode the antagonistic effects between CGA and retrorsine.

Comparing the effects of two forms of PAs in combination with plant fractions, we found retrorsine to be more toxic, which however is contingent on the chemical backgrounds in which it is presented. The order of activity of the two PAs is reversed when they are presented together with a high concentration of the *n*-hexane extract. The current findings and our previous results emphasize the complexity of the interactions between plant metabolites and their consequences for bioactivity.

### How Important Are Natural Backgrounds for the Bioactivity of Individual SMs?

Natural backgrounds are important for individual metabolites in affecting the pattern and the strength of bioactivity. Insect herbivores always encounter mixtures of metabolites in nature. Thus a given plant metabolite is likely not to be the sole agent, but rather is likely to be a participant in a multitude of interactions that naturally occur in plants. This study supports a commonly held notion that plant chemical defences are dependent on a variety of metabolites, which together shape the outcome of the defensive efficacy. Several subsets of metabolites generated through fractionation of the original extract differed in the strength of the interaction with retrorsine. This again indicates that the effects of plant metabolites vary with the chemical background in which they are present. This could explain, in some cases, why a single metabolite is active in one species while being less active or even inactive in other species. For instance, CGA in *Chrysanthemum* was negatively correlated with the feeding damage of WFT (Leiss et al. 2009), while no effect of CGA on WFT was detected in tomato *Solanum lycopersicum* (Mirnezhad 2011). Genetic modification approaches targeting single biosynthetic genes, have been successful in showing the effect of single metabolites in an otherwise unchanged chemical background (Voelckel et al. 2001).

Only for a few species and metabolites a genetic modification approach is feasible. A fruitful alternative is to spike fractions or extracts representing their natural chemical background with these metabolites. In the case of the PAs tested here, we see that activity of the PAs is indeed altered upon adding the natural chemical background. This proves to be a simple and straightforward method to make a better prediction of the activity of PAs in ecological relationships.

Our approach should be considered a first step to study the effect of interactions between plant metabolites on their bioactivity against WFT. Results from this initial step clearly indicated the importance of interactions between metabolites on WFT survival. Further sub-fractionation may narrow down the candidate compounds that are involved in these interactions. A challenge in the following component-interaction analysis is to measure the effects of the almost infinite number of possible combinations, due to the enormous number of metabolites in a plant. High-throughput screening, like the one used here, is essential for assaying the bioactivity of a large number of potential candidates against a chosen target. Another potential useful approach is to screen for anti-herbivore activities by using insect cell lines (Nuringtyas et al. 2014). However, also here it should be considered that cells function in the background of an organ, which is embedded in an entire organism.

## Electronic supplementary material

ESM 1 (DOCX 3360 kb)
