# Supplementary material for: Phytochemical Background Mediates Effects of Pyrrolizidine Alkaloids on Western Flower Thrips
Source: J Chem Ecol. 2018 Sep 16;45(2):116–27. doi: 10.1007/s10886-018-1009-2 (PMC6469620; doi:10.1007/s10886-018-1009-2)
Supplement: Supplementary file 1 — (DOCX 3360 kb) [file 10886_2018_1009_MOESM1_ESM.docx]

**PHYTOCHEMICAL BACKGROUND MEDIATES EFFECTS OF PYRROLIZIDINE ALKALOIDS ON WESTERN FLOWER THRIPS**

XIAOJIE LIU^1,2^ *, KLAAS VRIELING^1^, PETER G.L. KLINKHAMER^1^

*^1^ Plant Ecology and Phytochemistry, Institute of Biology, Leiden University, PO Box 9505, 2300 RA Leiden, The Netherlands*

*^2^ Modern Research Center for Traditional Chinese Medicine, Shanxi University, Taiyuan 030006, China*

* Corresponding author. E-mail: x.liu.2@biology.leidenuniv.nl

1. **Supplementary figures**

**Figure Legend**

**FIG. S1.** Experimental design of the *in vitro* thrips bioassay of one well of a 96 wells plate

**FIG. S2.** A schematic figure illustrating the repeation of the experiments

**FIG. S3.** Total PA concentration in the five fractions of *Jacobaea* shoots derived from the MeOH extract (mM) at the highest concentration tested. Data are based on the LC-MS/MS analysis of the fractions

**FIG. S4.** Log-transformed rates of 2^nd^ instar Western flower thrips (WFT) (*Frankliniella occidentalis*) against the amount of *Jacobaea* plant mass equivalents per mL of the methanol extract (**A**) and the five fractions of the methanol extract: *n*-hexane fraction (**B**), CHCl_3_ fraction (**C**), EtOAc fraction (**D**), *n*-BuOH fraction (**E**) and H_2_O fraction (**F**). WFT larvae were put on an artificial diet at five concentrations for 5 days. Survival rate was corrected for differences among negative controls. For significant regressions, lines are shown (see also Table 1 for the corresponding statistical tests)

**FIG. S5.** Log-transformed survival of 2^nd^ instar Western flower thrips (WFT) (*Frankliniella occidentalis*) against the concentrations of *n*-hexane fraction (open dots), CHCl_3_ fraction (solid triangles), EtOAc fraction (stars), *n*-BuOH fraction (solid dots) and H_2_O fraction (squares). Dots are averages ± SE (n = 4)

**FIG. S6.** Western flower thrips (WFT) survival rates of 5 fractions of a *Jacobaea* methanol extract, retrorsine and the combination of retrorsine with *n*-hexane fraction (**A**), CHCl_3_ fraction (**B**), EtOAc fraction (**C**), *n*-BuOH fraction (**D**) and H_2_O fraction (**E**)

**FIG. S7.** Survival rate of 2^nd^ instar larvae of Western flower thrips (*Frankliniella occidentalis*) of individual fractions of *Jacobaea* plant or retrorsine *N*-oxide and the combination of retrorsine *N*-oxide with *n*-hexane fraction (**A**), CHCl_3_ fraction (**B**), EtOAc fraction (**C**), *n*-BuOH fraction (**D**) and H_2_O fraction (**E**)

**FIG. S8.** High-performance thin-layer chromatographs (HPTLC) of the MeOH extract of *Jacobaea* shoots **(Track 1)**, five fractions derived from the MeOH extract **(Track 2-6),** the re-combined fraction **(Track 7)** and references compounds (**Track 8-12**). The TLC plate was detected after derivatisation with the natural products reagent (NPR) and PEG 4000 at 366 nm. **Plant fractions: Track 2:** the *n*-hexane fraction; **Track 3:** the CHCl_3_ fraction; **Track 4:** the EtOAc fraction; **Track 5:** the *n*-BuOH fraction; **Track 6:** the H_2_O fraction. **Reference compounds: Track 8:** rutin; **Track 9: c**hlorogenic acid; **Track 10:** quercetin; **Track 11:** caffeic acid; **Track 12:** ferulic acid

[
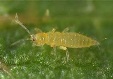
](http://www.google.com.hk/url?sa=i&rct=j&q=&esrc=s&frm=1&source=images&cd=&cad=rja&uact=8&docid=KPtHMutXXB3ZgM&tbnid=OvxxufN-IU6YdM:&ved=0CAUQjRw&url=http://www.agf.gov.bc.ca/cropprot/grapeipm/thrips.htm&ei=WmWPU9GLL8KyPLbQgdgL&psig=AFQjCNEUbz2P2gYmgVDjJtPv6nexOIYxZg&ust=1401992892342105)

The flat cap


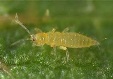


Test solution

Up-side down

Thrips larvae

Test solution

Parafilm^TM^

**FIG. S1.**


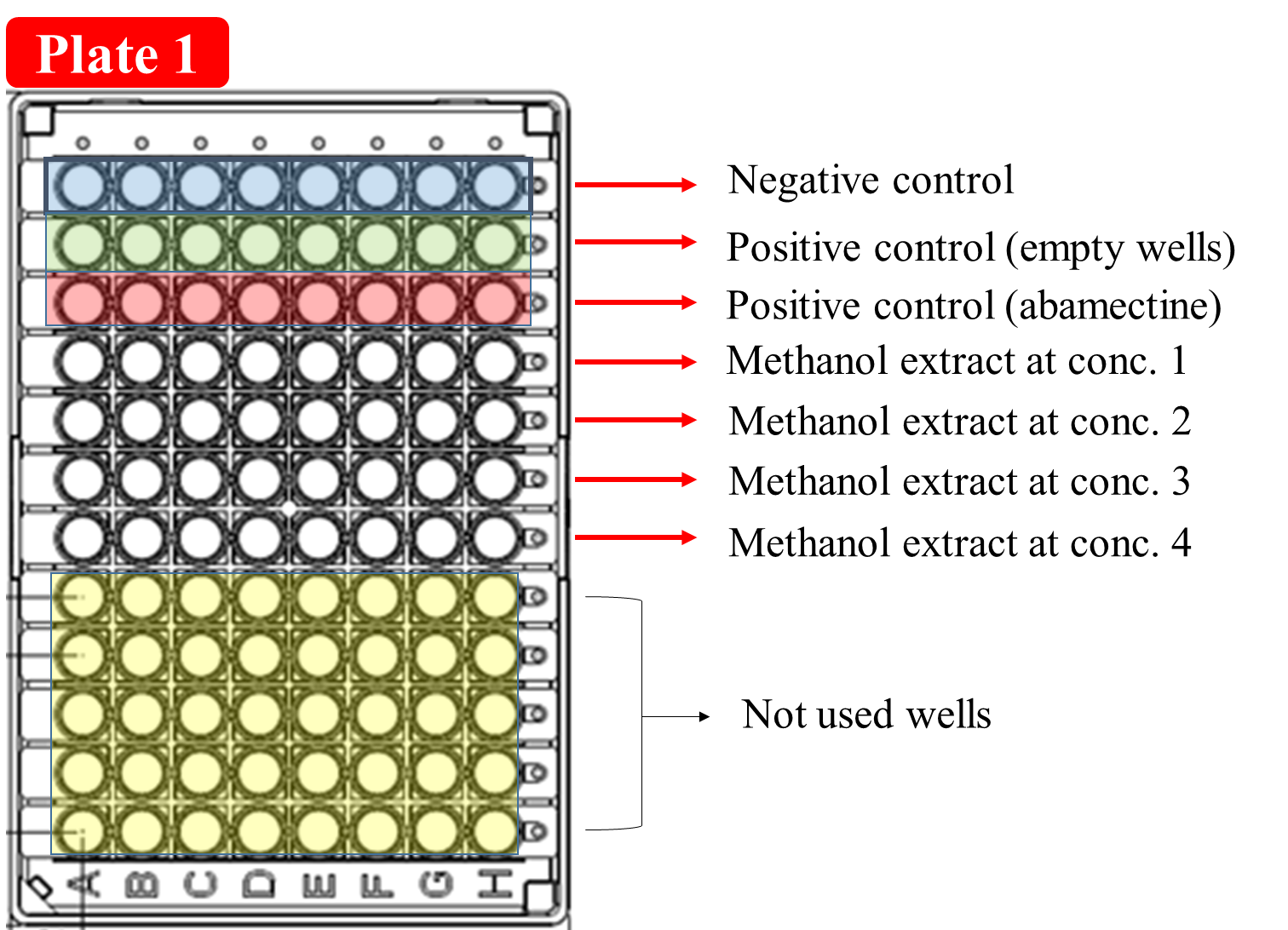


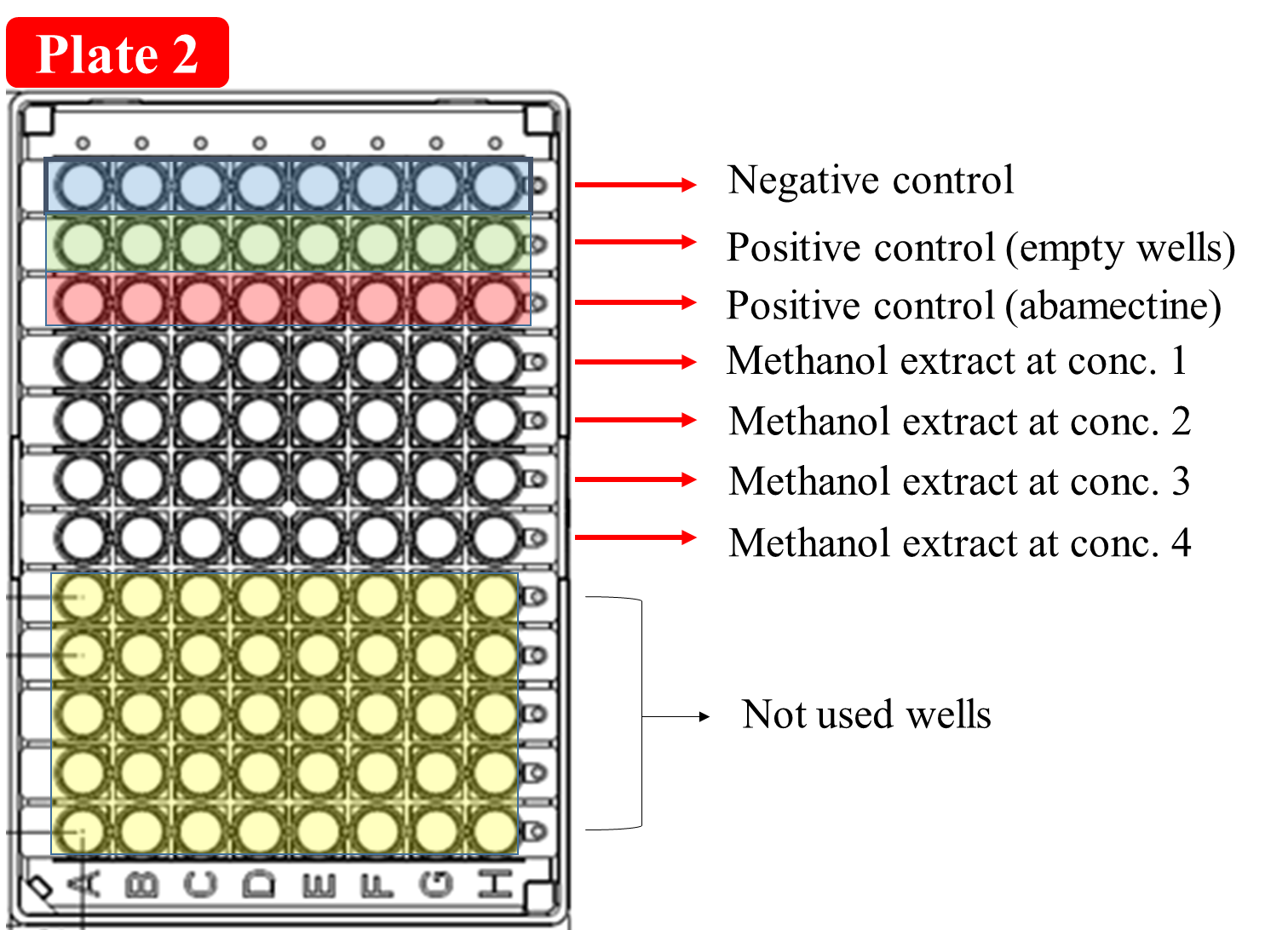


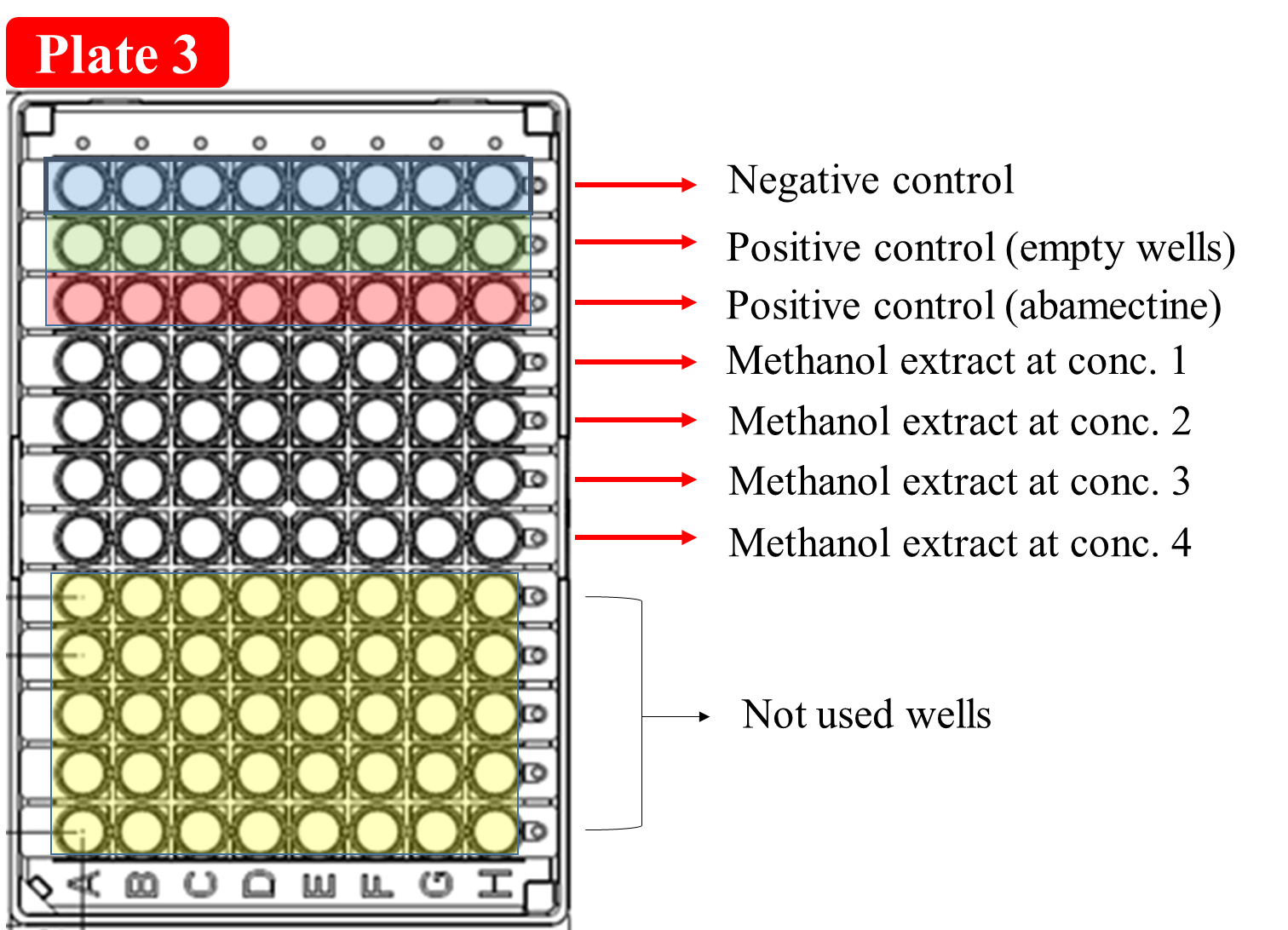


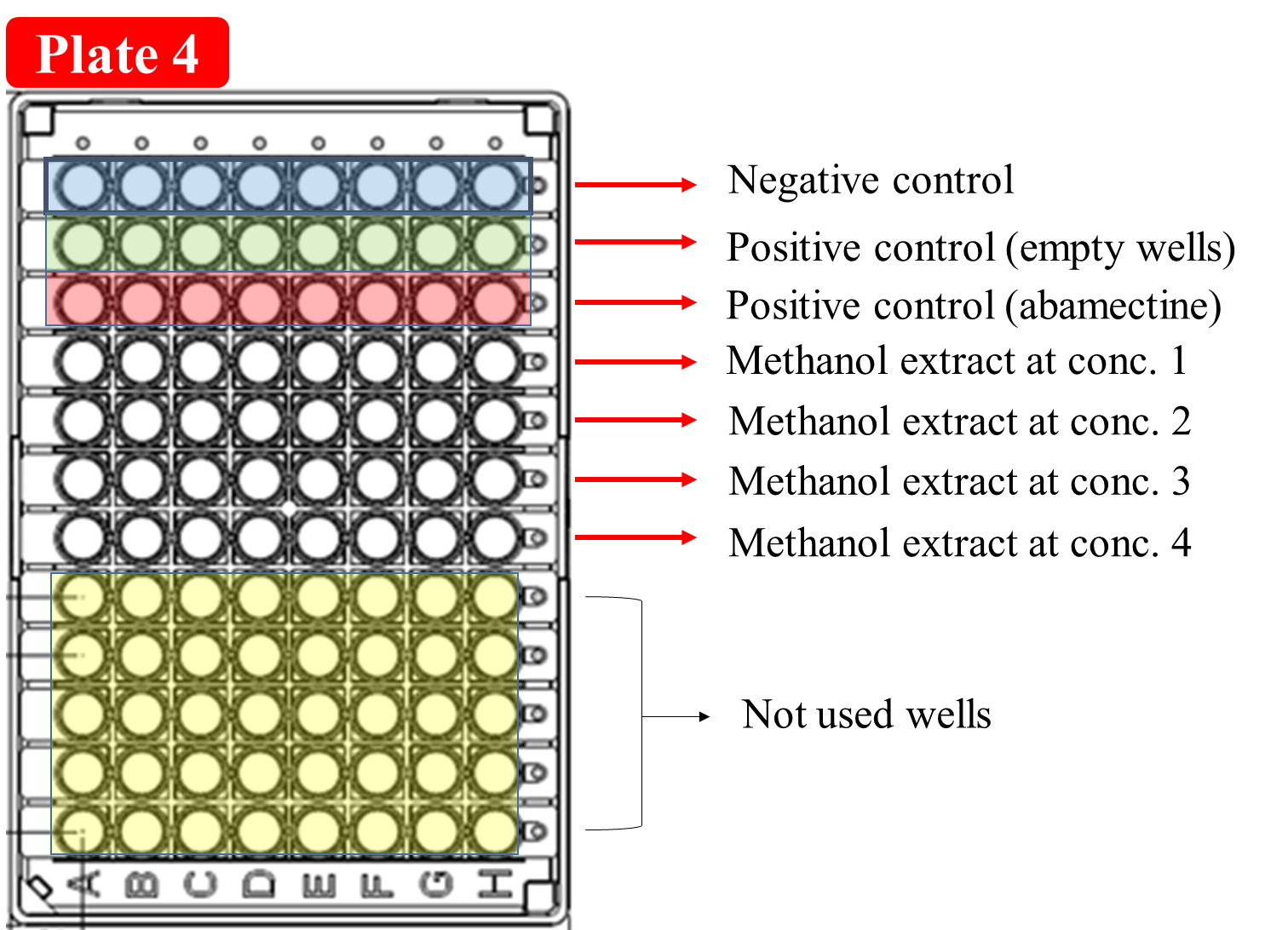


**FIG. S2**

**FIG. S3**

**Mass equivalents (g/mL)**

**Mass equivalents (g/mL)**

**Log WFT survival rates**

**Log WFT survival rates**

**Log WFT survival rates**

**FIG. S4**

**FIG. S5**

**FIG. S6**

**FIG. S7**


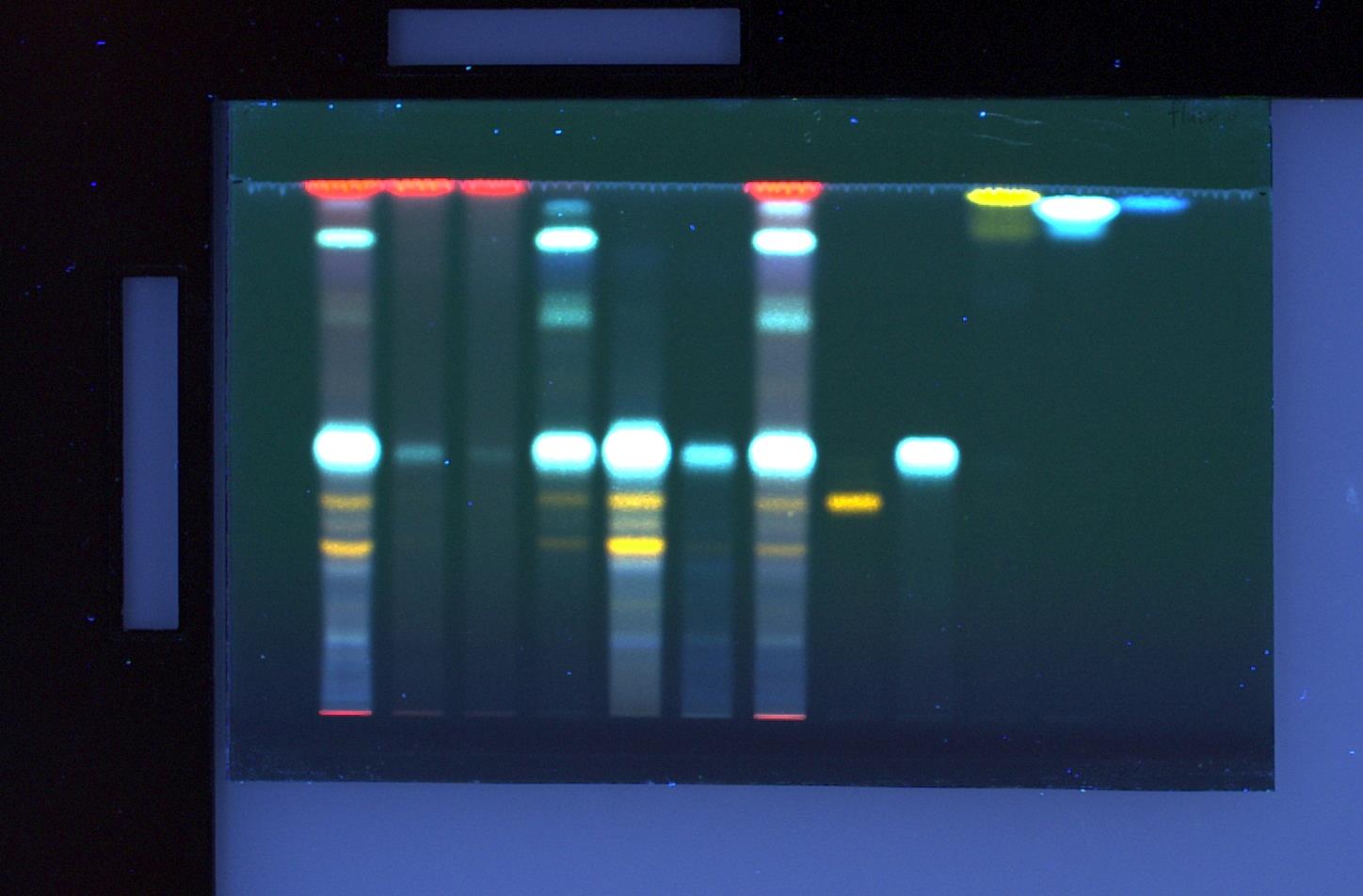


1 2 3 4 5 6 7 8 9 10 11 12

**FIG. S8**

1. **Supplementary tables**

**TABLE S1.** Two-way analyses of variance (ANOVAs) with fraction concentration and retrorsine concentration as fixed factors and the interaction effect S_PA*F_ minus one as the dependent variable. A significant intercept indicates a synergistic or antagonistic interaction

| **Factors** | ***df*** | ***F*** | ***P*** |
| --- | --- | --- | --- |
| Intercept | 1, 11 | 23.9 | <0.01 |
| *n*-hexane fraction concentration | 2, 11 | 1.1 | NS |
| Retrorsine concentration | 1, 11 | 4.3 | NS |
| *n*-hexane fraction concentration * Retrorsine concentration | 2, 11 | 1.9 | NS |
| Intercept | 1, 11 | 3.1 | NS |
| CHCl_3_ fraction concentration | 2, 11 | 0.9 | NS |
| Retrorsine concentration | 1, 11 | 7.2 | <0.05 |
| CHCl_3_ fraction concentration * Retrorsine concentration | 2, 11 | 0.5 | NS |
| Intercept | 1, 11 | 0.4 | NS |
| EtOAc fraction concentration | 2, 11 | 1.1 | NS |
| Retrorsine concentration | 1, 11 | 0.1 | NS |
| EtOAc fraction concentration * Retrorsine concentration | 2, 11 | 0.4 | NS |
| Intercept | 1, 10 | 37.0 | <0.01 |
| *n*-BuOH fraction concentration | 2, 10 | 0.7 | =0.06 |
| Retrorsine concentration | 1, 10 | 5.8 | NS |
| *n*-BuOH fraction concentration * Retrorsine concentration | 2, 10 | 0.7 | NS |
| Intercept | 1, 11 | 9.6 | <0.05 |
| H_2_O fraction concentration | 2, 11 | 7.5 | <0.05 |
| Retrorsine concentration | 1, 11 | 0.1 | NS |
| H_2_O fraction concentration * Retrorsine concentration | 2, 11 | 0.3 | NS |

NS = Not significant

**TABLE S2.** Two-way analyses of variance (ANOVAs) with fraction concentration and retrorsine *N*-oxide concentration as fixed factors and the interaction effect S_PA*F_ minus one as a dependent variable. A significant intercept indicates a synergistic or antagonistic interaction

| **Factors** | ***df*** | ***F*** | ***P*** |
| --- | --- | --- | --- |
| Intercept | 1, 11 | 33.9 | < 0.001 |
| *n*-hexane fraction concentration | 2, 11 | 4.4 | NS |
| Retrorsine *N*-oxide (Re NO) concentration | 1, 11 | 0.03 | NS |
| *n*-hexane fraction concentration * Re NO concentration | 2, 11 | 0.3 | NS |
| Intercept | 1, 11 | 127.8 | < 0.001 |
| CHCl_3_ fraction concentration | 2, 11 | 3.2 | NS |
| Re NO concentration | 1, 11 | 15.8 | < 0.01 |
| CHCl_3_ fraction concentration * Re NO concentration | 2, 11 | 4.1 | NS |
| Intercept | 1, 11 | 0.5 | NS |
| EtOAc fraction concentration | 2, 11 | 5.3 | < 0.05 |
| Re NO concentration | 1, 11 | 0.005 | NS |
| EtOAc fraction concentration * Re NO concentration | 2, 11 | 0.06 | NS |
| Intercept | 1, 11 | 21.5 | < 0.01 |
| *n*-BuOH fraction concentration | 2, 11 | 0.3 | NS |
| Re NO concentration | 1, 11 | 6.4 | < 0.05 |
| *n*-BuOH fraction concentration * Re NO concentration | 2, 11 | 0.4 | NS |
| Intercept | 1, 11 | 2.0 | NS |
| H_2_O fraction concentration | 2, 11 | 2.6 | NS |
| Re NO concentration | 1, 11 | 0.2 | NS |
| H_2_O fraction concentration * Re NO concentration | 2, 11 | 0.1 | NS |

NS = Not significant
